# Predicting risk of malignancy in subgroups of B3 breast lesions

**DOI:** 10.1186/bcr3312

**Published:** 2012-11-09

**Authors:** ND Forester, M Brotherton, A-M Wason

**Affiliations:** 1 Department of Breast Radiology, Bradford, UK

## Introduction

Heterogeneity and varying malignancy risk makes B3 lesion management difficult. Can histological features predict malignancy risk?

## Methods

A retrospective review of B3 lesions (April 2005 to March 2010) following 14G biopsy, followed to final pathology. Key phrases from pathology identified; atypia, radial scar/complex sclerosing lesion (RS/CSL), atypical intraductal proliferation (AIDP), atypical ductal/lobular hypertrophy (ADH/ALH), flat epithelial atypia (FEA), lobular *in situ *neoplasia (LISN), and so forth. Age-adjusted logistic regression to assess risk of malignancy (Stata11).

## Results

A total of 205 B3 lesions were identified; 112 lesions with subsequent excision biopsy were analysed. Patients had mean age of 56 years (95% CI = 55 to 57 years). Thirty out of 112 lesions were upgraded to B5 diagnosis. Nine out of 112 had final diagnosis of LCIS. Multivariate analysis of odds ratios for malignancy, after age adjusting, is shown in Table 1.

**Table 1:** 

Pathology	Number	Odds ratio	*P *value	95% CI
Atypia	53	7.48	< 0.001*	2.62 to 21.39
AIDP	10	4.87	0.034*	1.13 to 21.01
ADH	11	2.50	0.167	0.68 to 9.24
ALH	6	1.28	0.793	0.21 to 7.93
FEA	8	5.90	0.025*	1.25 to 27.67
CCC	31	5.10	0.001*	1.89 to 13.76
Papillary	14	0.57	0.44	0.14 to 2.38
LISN	30	0.55	0.28	0.18 to 1.64
RS/CSL	13	0.92	0.91	0.23 to 3.74
Epithelial proliferation	20	2.07	0.172	0.73 to 5.88
Mucocele-like	6	1.91	0.48	0.32 to 11.41
Fibroepithelial	10	No malignant diagnoses		
Haemangioma	1	No malignant diagnoses		
Spindle cell	2	No malignant diagnoses		

*Significant *P *value.

## Conclusion

Atypia on core biopsy significantly predicts malignancy, with 7.48 times the odds of malignancy compared with lesions without atypia (*P *< 0.001; 95% CI = 2.62 to 21.39). Similarly, lesions containing AIDP, FEA and columnar cell change (CCC) have significantly increased odds for malignancy. LISN did not confer an increased risk of malignancy. Stratifying lesions in this way can direct future management.

